# Adaptive perceptual responses to asymmetric rotation for testing otolithic function

**DOI:** 10.1007/s00221-022-06393-9

**Published:** 2022-06-18

**Authors:** Mario Faralli, Chiara Pelliccia, Chiara Occhigrossi, Rosa Bruni, Francesco Frati, Giampietro Ricci, Vito Enrico Pettorossi

**Affiliations:** 1 Department of Medicine e Surgery, Otorhinolaryngology Section, Università degli Studi di Perugia, Piazzale Gambuli, 1, 06129 Perugia, Italy; 2 Department of Medicine and Surgery, Human Physiology and Biochemistry Section, Università degli Studi di Perugia, Piazzale Gambuli, 1, 06129 Perugia, Italy

**Keywords:** Self-motion perception, Otolithic function, Gravity, Perceptual adaptation, Head position

## Abstract

This study aimed to test the role of the otolithic system in self-motion perception by examining adaptive responses to asymmetric off-axis vertical rotation. Self-movement perception was examined after a conditioning procedure consisting of prolonged asymmetric sinusoidal yaw rotation of the head on a stationary body with hemicycle faster than the other hemicycle. This asymmetric velocity rotation results in a cumulative error in spatial self-motion perception in the upright position that persists over time. Head yaw rotation conditioning was performed in different head positions: in the upright position to activate semicircular canals and in the supine and prone positions to activate both semicircular canals and otoliths with the phase of otolithic stimulation reversed with respect to activation of the semicircular canals. The asymmetric conditioning influenced the cumulative error induced by four asymmetric cycles of whole-body vertical axis yaw rotation. The magnitude of this error depended on the orientation of the head during the conditioning. The error increased by 50% after upright position conditioning, by 100% in the supine position, and decreased by 30% in the prone position. The enhancement and reduction of the perceptual error are attributed to otolithic modulation because of gravity influence of the otoliths during the conditioning procedure in supine and prone positions. These findings indicate that asymmetric velocity otolithic activation induces adaptive perceptual errors such as those induced by semicircular canals alone, and this adaptation may be useful in testing dynamic otolithic perceptual responses under different conditions of vestibular dysfunction.

## Introduction

Otolithic receptors contribute to vestibular reflexes and perception of head position and movement signaling linear head acceleration. The interaction of otoliths with the semicircular canals has been shown by examining angular and linear vestibulo-ocular reflex (VOR) during off-vertical axis rotation and linear and angular translation (Harris 1988; Furman et al. 1992; Crane and Demer 1997; Jaggi-Schwarz et al. 2000). In addition, the role of the otoliths and canals in self-motion perception has largely been examined in response to translation and rotation in the horizontal plane (Israel et al. 2005; Ivanenko et al. 1997; MacNeilage et al. 2010; Soyka et al. 2015; Crane 2016; Merfeld et al. 1999). For clinical evaluation of the otolithic reflex, vestibular evoked myogenic potentials (VEMPs) (Colebatch et al. 1994; Curthoys et al. 2011), ocular cyclotorsion (Halmagyi 1979; Diamond and Markham 1983; Lapenna et al. 2018) and translational VOR (LVOR) (Gresty and Bronstein 1992) have been tested. Conversely, the role of otoliths in the perceptual dynamic responses has been poorly investigated in clinical examination. Indeed, the perceptual test for otoliths is the study of subjective visual vertical (SVV) (Bohmer and Rikenmann 1995; Faralli et al. 2007), but this test is commonly used for assessing static otolithic perceptual function.

The reason for this poor investigation in patients may be the complexity of instrumentation needed for otolithic stimulation such as linear slides or special motion platforms.

Nevertheless, the study of dynamic perceptual otolithic responses appears relevant because the otolithic receptors responsible for transient responses may differ from tonic receptors. In addition, ocular reflex responses do not necessarily reflect perceptual function because perception and reflex show differences due to different central processing and adaptive processes. Several studies on the vestibular system have examined the perception of self-movement in response to body rotation activating semicircular canals. These studies showed similarities and differences in immediate and adaptive responses between eye reflex and motion perception (Mergner and Rosemeier 1998; Seemungal et al. 2004; Merfeld et al. 2005; Cousins et al. 2013; Bertolini et al. 2011; Pettorossi et al. 2013, Anagnostou et al. 2018).

In recent studies, responses to repetitive asymmetric stimulation demonstrated adaptive opposite behavior between reflex and perceptual responses (Pettorossi et al. 2013). In that study, the VOR and self-motion perception in response to repetitive asymmetric horizontal rotation were associated with a different adaptation to sinusoidal rotation, which is faster in one direction compared with the other (asymmetric rotation). In fact, the perception of slow rotation decreased significantly, whereas the perception of fast rotation increased slightly after a few cycles of repetition. In contrast, the VOR showed no adaptation. The discrepancy between reflex and perceptual adaptive responses has also been demonstrated in patients with vestibular neuritis, where compensation for the perceptual imbalance was much slower than for the VOR in response to asymmetric stimulation (Panichi et al. 2017a, b).

Because of the different central processing of reflex and perception, the analysis of reflexes alone cannot exhaust functional investigations of the vestibular system. This suggests that it is necessary to also include motion perception in otolithic function examination.

This study aimed to provide evidence for the contribution of the otolithic system to motion perception and its adaptation by testing perceptual responses to yaw whole-body rotation after prior conditioning of repetitive yaw asymmetric head rotation in different head positions, including or excluding otolithic stimulation during rotation. Repetitive asymmetric rotation conditioned the vestibular responses by altering perception, mainly by reducing the perceived velocity of slow rotation. Vertical axis yaw rotation (head in the upright position) engages the semicircular canals alone, while off-vertical axis rotation (head in supine and prone positions) engages both semicircular canals and otoliths. Conditioning at different head positions may allow assessment of the exclusive contribution of the semicircular canals and the combination of both canal and otolithic stimulation in self-motion perception. In addition, conditioning in supine and prone positions may provide further information since otolithic activation is reversed with respect to that of the semicircular canals (Harris 1988; Pettorossi et al.^1997^). After these different conditioning procedures, the perception of self-movement was tested by vertical axis whole-body rotation in the upright position. The perceptual difference shown by this asymmetric conditioning test may reveal the contribution of the otolithic system to motion perception in normal rotation.

## Methods

Sixteen healthy individuals, aged between 20 and 45 years (10 men, 6 women; mean age 25.8 years), participated in this study after providing written informed consent. The experimental protocol complied with the Declaration of Helsinki (1964) and was approved by the Ethics Committee of the University of Perugia. All individuals were right-handed and their hearing was within normal limits. Testing of individuals was performed by a subgroup of the authors, which was different from the subgroup who performed the data analysis, and who were not aware of the research questions.

### Vestibular stimulation

The procedure for vestibular stimulation to test self-motion perception has been reported in earlier studies (Pettorossi et al. 2013; Panichi et al. 2017a, b; Faralli et al. 2021). The participants were tested in an acoustically isolated cabin and sitting in a computer-controlled chair that could be rotated in the horizontal plane. Using a video camera, the motion of the head, secured to the chair, was verified by recording the displacement of two pairs of reflective infrared markers placed on the head. The trial was discontinued when the head markers revealed occasional displacements unrelated to the stimulus.

**Fig. 1:**
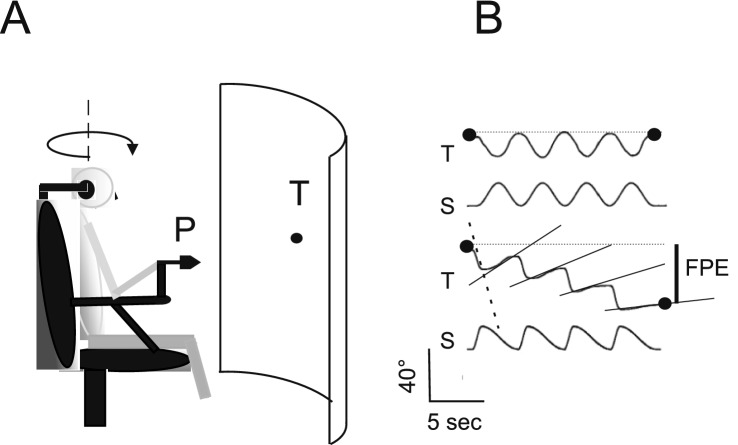
**A** Experimental setting for the self-motion perception testing. The subject seats on rotatory chair and maneuvers the pointer (P) to pursuit the remembered visual target (T) presented before the rotation. The head is pitched 30° nose down and the rotation is performed in the horizontal plane (arrow). **B** Target tracking during symmetric and asymmetric rotation. The tracking during symmetric sinusoidal rotation (upper two traces) and during asymmetric sinusoidal rotation (lower two traces) (T, tracking; S, stimulus) are reported. The black spots represent the initial and the final position of the target representation. Below: tracking calibration. Note that the final position of the target representation is correct after symmetric rotation, since no spatial disparity is present between the initial and final position. Conversely, the target representation is erroneous after asymmetric rotation as shown by the disparity of the initial and final position (FPE, final position error). The inclined lines, fitting the tracking during slow rotation, show the progressive reduction of slow-motion perception during asymmetric rotation

The chair oscillated asymmetrically in the dark with half-sinusoidal cycles at different speeds (Fig. 1). The individuals were rapidly rotated to one side and then slowly returned to the original position [same amplitude (40°) but different frequencies: fast half-cycle (FHC) = 0.38 Hz, peak acceleration 120°/s^2^, peak velocity 47°/s; slow half-cycle (SHC) = 0.09 Hz, peak acceleration 7°/s^2^, peak velocity 11°/s]. Both rotational velocities were supra-threshold for vestibular stimulation. The rotation was repeated four times (Fig. 1).

In a subsequent experimental period 1 day later, an additional study was performed by symmetric rotation at 0.1 Hz (4 cycles; peak acceleration 7.9°/s^2^, peak velocity 12.5°/s) to show the different perceptual effects compared with asymmetric rotation.

### Recording self-motion perception

We used a psychophysical tracking procedure to assess perception of self-movement (Pettorossi et al. 2004; Siegle et al. 2009). Before starting the rotation, the participants were asked to look at a target placed in front of them and to imagine the target during rotations performed in the dark and with their eyes closed. The target was represented by a bright spot (1 cm in diameter) projected onto a cabin wall at eye level (Fig. 1). The spot was switched off immediately before the start of rotation. Individuals were instructed to continuously follow the memorized spot in the dark by rotating a hand pointer toward that spot. The pointer, connected to a precision potentiometer pivoted on a stand attached to the chair, allowed continuous recording of the angular displacement. In addition, the pointer had a laser beam that was switched on at the end of the rotation to measure the angular distance between the projection of the beam onto the wall and the light that was switched on. The pointer and chair movement signals were digitized using a 12-bit analog–digital board (LabVIEW, National Instruments, Austin, TX, USA) at a sampling rate of 500 Hz and stored for offline analysis. The remembered target position was evaluated directly by measuring the position of the laser beam on the wall and the actual target position, whereas manual tracking was recorded using the pointer position signal to verify the quality of self-movement during asymmetric rotation. The tracking response was considered acceptable when it resembled the sinusoidal profile of the stimulus. Individuals were trained to track the remembered target, initially in the presence of visual feedback provided by the laser beam of the pointer, and thereafter, without the feedback, and only tracking was recorded. The experimenter evaluated the quality of tracking during the training and testing periods by observing the potentiometer signal. Tracking periods with response discontinuities, jerks, or pauses were discarded.

### Testing self-motion perception in response to asymmetric rotation

Because of the vestibular system’s characteristic transfer function and additional adaptive responses, the participants perceived the FHC more vividly than the SHC (Pettorossi et al. 2013; Panichi et al. 2017a, b). Therefore, the different motion perceptions during the fast and slow half-cycles induced misrepresentation of the remembered target position. This contrasting velocity rotation-induced adaptive reduction in slow-motion perception and a slight enhancement in fast rotation perception resulted in a persistent cumulative error in self-motion perception. Because of this cumulative error, at the end of the four-cycle asymmetric session, the target was represented in an erroneous position (final position error, FPE) laterally with respect to the real target position in the direction opposite to that of the FHC (Fig. 1). The test was performed five times at intervals of more than 15 min, and the mean value of the FPE was reported for each participant to verify the effect of the conditioning procedure.

### Conditioning procedure

**Fig. 2:**
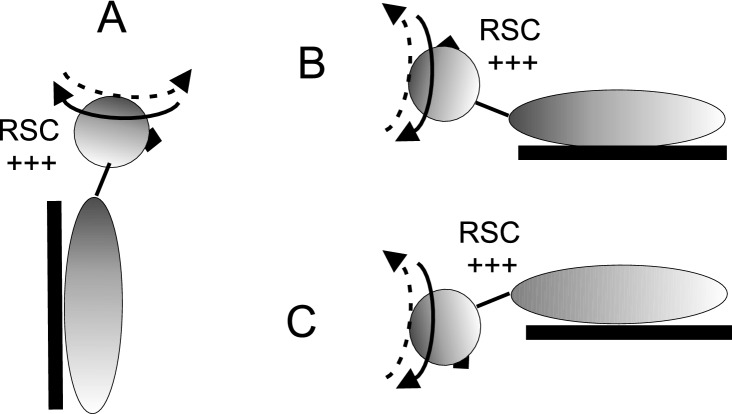
Head position in three conditioning head position: upright (**A**), supine (**B**) and prone (**C**). The head is quickly rotated toward one side (solid arrow) and slowly rotated back to the center (dashed arrow). In all positions the direction of fast rotation is toward the right horizontal semicircular canals (RSC) and induces an intense right canal activation. Conversely, the fast dynamic influence of the gravity vector on the otolithic receptors is different during asymmetric rotation: the otolithic receptors are not influenced by gravity in (**A**) and they are activated in the other two cases, but the direction of the dynamic modulation of otolith and canal is opposite in supine (**B**) compared with prone (**C**) position

Individuals were conditioned by asymmetric yaw head rotation on a stationary body in three different positions: upright, supine, and prone (Fig. 2). In all conditioning procedures, the individuals kept their eyes closed.

A preliminary study was performed to determine how many cycles of asymmetric rotation were necessary to condition self-motion perception by modifying the testing FPE in response to four cycles of whole-body rotation. Various cycles of prolonged asymmetric conditioning of head yaw rotation (4, 8, and 16 cycles) were used, all in the upright body position. Thereafter, conditioning at different head rotations in upright, supine, and prone positions was performed randomly at intervals of more than 1 h, to avoid any carry-over effect, and using the 16 cycle option which was the most efficient.

After preliminary study, 16 cycles of asymmetric head rotation were performed manually by the experimenter with both hands placed on the parietal skull.

**Fig. 3:**
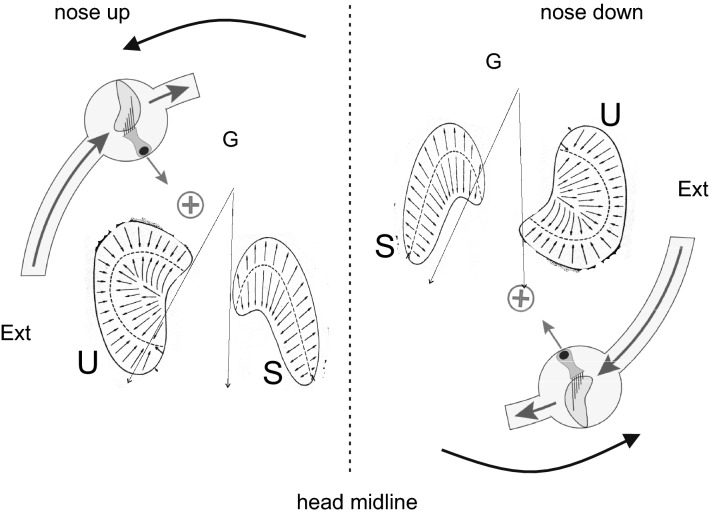
Activation of the horizontal semicircular canal and otolithic receptors during yaw rotation in supine (nose up, left side) and prone (nose down, right side) head position. In nose up and nose down the direction of the fast rotation (solid arrow) is the same for the horizontal semicircular canal, whereas the direction of fast activation by gravity is opposite for the otolithic receptors. U: Utricle, S: Sacculus. In nose up the rotation of the gravity vector displaced the otoliths toward the external side of the head (the arrow shift to the Ext), while in node down the gravity rotation displaces the otoliths toward the internal side (the arrow shift to Int). Vertical dashed line represents the head midline

The rotation was 40° from the center to one side; rotation away from the center lasted for 1 s (fast rotation) and rotation toward the center for 4 s (slow rotation). To move the head asymmetrically, the experimenter was assisted by an in-ear metronome (set at 60 bpm to count the duration of the rotation) and a laser affixed to the participant’s head, which projected the laser beam onto the wall to control the profile of head movement. The start and end periods of rotation were performed with a smoothly increasing and smoothly decreasing motion, respectively. Conditioning stimulations were performed with the head always tilted 30° nose down from the normal head position to align the horizontal semicircular canals with the horizon in the upright position, and with the chin 30° towards the chest, on the subsequent day downwards relative to the long axis of the body in the prone and supine positions. The motion of endolymph in the lateral semicircular canals and the direction of otolithic displacement induced by gravity in the supine and prone positions are shown in Fig. 3.

### Experimental procedure

Two minutes after each conditioning procedure (16 cycles of head rotation alone for supine, prone, or upright positions), the individuals were placed on a rotatory chair with head tilted 30° nose down. Their self-motion perception was tested using four asymmetric cycles of whole-body yaw rotation in the upright position to establish the influence of the conditioning procedure on the amplitude of the FPE. The direction of fast rotation in the testing procedure was always the same as that in the conditioning procedure.

The tests with asymmetric head conditioning were performed on the same day while those with symmetric rotation were performed on the subsequent day. The time interval between each period of conditioning was about 10 min, an interval that, in our experience, was sufficient to avoid any carry-over effect. With some individuals, we changed the order of the position sequence and no difference in effects was observed. The experimental period for testing conditioning in three different positions lasted for 35 min. In this pivotal study, this 35-min test period was repeated five times to achieve greater statistical power.

### Statistical analysis

The data analyzed were the difference between the end of tracking and the target light (FPE), and we computed the mean values of the FPE from five tests per conditioning orientation per person.

Perception of self-movement was analyzed statistically using generalized linear mixed model (GLMM) analysis. We used the average value obtained from five different tests performed in each condition (whole body in upright, supine, and prone positions). Post hoc statistical tests were performed using Bonferroni correction for multiple comparisons. The significance level was set at *p* < 0.05 for both GLMM values and post-hoc comparisons. Before GLMM, the Shapiro–Wilk test (Shapiro and Wilk 1965) confirmed the normality of the distributions, and Levene's test confirmed the homogeneity of the variances of the pairs of distributions. The power values for all GLMM analyses are reported as η. All statistical evaluations were performed using OriginPro (Origin Lab Corporation, Northampton, MA, USA) and SPSS 16.0 (IBM Corp., Armonk, NY, USA).

## Results

### Perceptual testing procedure: self-motion perception in response to horizontal whole-body rotation

**Fig. 4:**
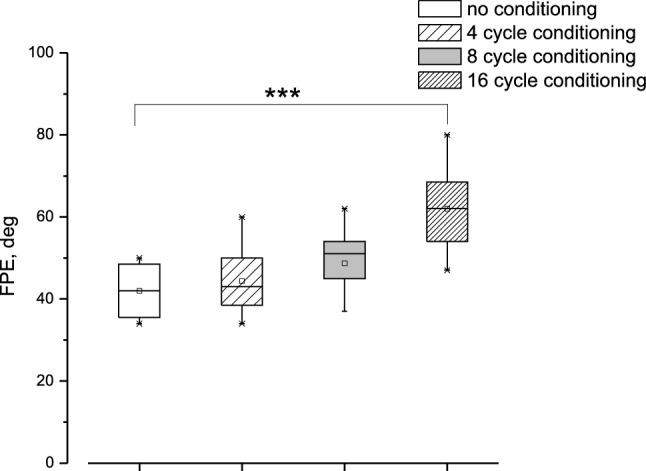
Magnitude of the FPE after conditioning with different numbers of head asymmetric yaw rotation in upright position. The FPE was induced by 4 cycles of whole-body asymmetric yaw rotation in upright position. Box, whisker, horizontal line and square represent range, quartile, median and mean, respectively. In abscissa the cycles of conditioning. FPE without prior conditioning (0) and after different yaw head asymmetric conditioning: 4 cycles, (4); 8 cycles (8), 16 cycles (16). Note that only the FPE after 16 cycles conditioning is significantly increased compared with that without conditioning (***p < 0.001)

**Fig. 5:**
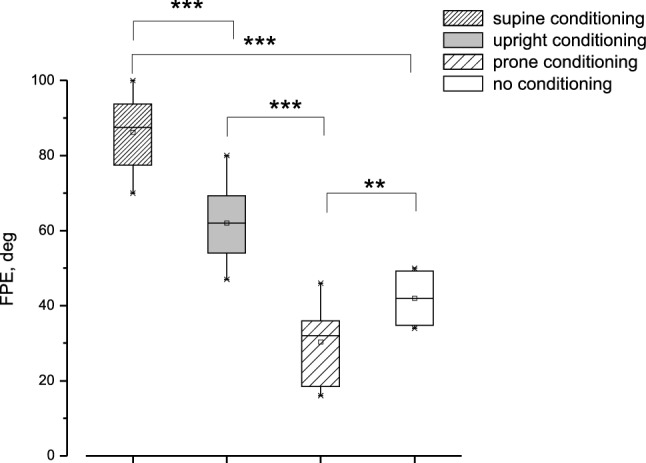
Conditioning effect of yaw asymmetric head rotation at different head positions on FPE induced by 4-cycle whole-body asymmetric yaw rotation. Box, whisker, horizontal line and square represent range, quartile, median and mean, respectively. In abscissa the conditioning position. The FPE observed without conditioning (non-conditioning) and the FPE after conditioning in supine (supine), upright (upright) and prone (prone) position. Note FPE significantly increases after conditioning in supine compared to the FPE after conditioning in upright position (***p < 0.001) and to the FPE without conditioning (***p < 0.001). Moreover, the FPE after prone conditioning is significantly lower than that observed without conditioning (**p < 0.01)

During four cycles of asymmetric horizontal whole-body rotation, all individuals showed a progressive shift in the reproduction of the remembered target position relative to the real position in the direction of slow hemicycle rotation. The amplitude of the target pursuit gradually diminished during the slow hemicycle response and was almost zero in the 4th cycle. In contrast, the amplitude of the target reproduction increased slightly during the fast hemicycle (Fig. 1B). Therefore, the target representation at the end of the cycles was erroneous and displaced in the opposite direction from the fast stimulus (final position error, FPE). The mean value of FPE (± SD) after the testing procedure was 42 ± 9° (Figs. 1, 4, 5). This value was regarded as the control value for comparing the effects of different conditioning procedures.

### Perceptual effect of asymmetric yaw head rotation conditioning in an upright body position (0° vertical rotation axis): a study to identify the number of cycles required to effectively condition perceptual responses

Various cycles of prolonged asymmetric conditioning of head yaw rotation (4, 8, and 16 cycles) were used, all in the upright body position, and the FPE in response to testing 4-cycle asymmetric whole-body yaw rotation in the upright position was examined. The FPE increased from 42 ± 9° to 44.5 ± 7° after 4 cycles of head rotation, to 48.7 ± 12° after 8 cycles, and to 62 ± 8° after 16 cycles (Fig. 4). The increase in the FPE was statistically significant (F (3,63) = 16.8, *p* < 0.001, η = 0.98), but post-hoc tests showed that the increase was only significant after 16 cycles (4 cycles: *p* = 0.89; 8 cycles: *p* = 0.22; 16 cycles: *p* < 0.001), showing an increase of about 50% (Fig. 4). Since the conditioning effect was only significant after 16 cycles, we used this number of cycles to compare conditioning in the upright position versus supine and prone conditioning (Fig. 5).

### Comparison of self-motion effects after conditioning in the upright, supine, and prone positions

The conditioning procedure for 16 cycles of asymmetric head yaw rotation was performed in all participants with a 90° tilted rotation axis (supine position) and a 270° tilted rotation axis (prone position), and the conditioning effects were compared with those induced by 0° rotation axis conditioning and without any conditioning (Fig. 3). The FPEs for all conditions were significantly different (F(3,60) = 120.9, *p* < 0.001, η = 0.91) compared to no conditioning (42 ± 9°) (*p* < 0.001) (Fig. 5). In the supine position, the mean FPE increased significantly by approximately 35% to 86 ± 8° (*p* < 0.001) with respect to upright conditioning (62 ± 8°) (*p* < 0.001) and 100% with respect to unconditioned FPE. In prone position conditioning, the FPE decreased significantly by approximately 45% to 29 ± 9° with respect to upright conditioning (*p* < 0.001) (Fig. 5). This value was significantly lower (by approximately 30%) than that observed in the unconditioned position (*p* < 0.01).

The dynamic perceptual conditioned responses of the otolithic system were revealed by comparing the FPE obtained in the upright position of the head with that induced in the supine position for each participant. The difference in value suggests that there was a contribution from otolithic modulation by gravity during conditioning. The difference was significantly greater (F (2,39) = 29.7, *p* < 0.001, η = 0.89; post-hoc: *p* < 0.001) when the response after supine conditioning was compared with that without conditioning. In this case, the contribution to the change in FPE was due to both otolithic and canal conditioning.

To confirm the usefulness of asymmetric testing, 4 cycles of symmetric rotation were delivered to the participants to observe the effect of perceptual self-motion. The symmetric rotation was not able to reveal difference in the representation of the remembered target after asymmetric conditioning in upright, supine and prone head positions. In fact, the error in the representation of the remembered target was not significantly different among that observed in three conditioning positions (F(2,47) = 3.9, *p* > 0.7, η = 0.80).

## Discussion

In this study, it was shown that self-motion perception is conditioned by prior asymmetric head rotation in prone and supine positions which activates the semicircular canals and otoliths. This conditioning not only confirms that otoliths contribute to the central reconstruction of movement perception during off-axis vertical rotation, as shown in previous studies (Ivanenko et al. 1997; Merfeld et al. 1999; Israel et al. 2005; MacNeilage et al. 2010; Soyka et al. 2015; Carriot et al. 2015; Crane 2016), but also shows that the signal from otolithic activation adapts in response to asymmetric dynamic activation in a similar way to that of the signals from the semicircular canals (Pettorossi et al. 2013). The adaptive responses to asymmetric rotation were necessary to reveal the otolithic influence, since symmetric rotation did not reveal any sizeable distinct effect.

### Semicircular canal contribution to self-motion perception in response to asymmetric yaw head rotation in the upright position (vertical rotation axis 0°)

The FPE resulting from the application of four asymmetric whole-body rotations was influenced by earlier asymmetric head rotation. In fact, conditioning by rotation with vertical axis yaw head rotation induces a higher FPE than observed without conditioning. Several asymmetric head rotations are required to achieve a significant effect, more than could be expected from whole-body rotation (Pettorossi et al. 2013). This is conceivable because asymmetric head oscillation alone includes both vestibular and neck proprioceptive stimulation. Because the asymmetric sinusoidal rotation has a different velocity of hemicycles but the same amplitude, it is likely that the proprioception of the neck muscles, which also provides position signals in addition to velocity, could attenuate the disparity of the hemicycles of the stimulus (Mergner et al. 1992). This may likely reduce rotation misperception during slow rotation and the conditioning effect of asymmetric stimulation.

### Otolithic contribution to self-motion perception in response to asymmetric yaw head rotation in the supine (vertical rotation axis 90°) and prone (vertical rotation axis 270°) positions

To highlight the role of the otolithic signal in the modulation of self-movement perception, we studied the amplitude of the FPE induced by four cycles of asymmetric whole-body rotation after conditioning to different axes of head rotation during the conditioning procedure. The effect of conditioning on the FPE was significantly different depending on the head position during yaw rotation conditioning, resulting in the presence or absence of combined activation of semicircular canals and otoliths. In the upright position, rotation of the vertical axis activates only the semicircular canals, whereas in the supine and prone positions, otolithic receptors are also activated as the gravity vector displaces the otolithic masses during rotation (Figs. 2, 3). The inclusion of otolithic conditioning significantly changes the effect of conditioning by increasing or decreasing the effect observed using only semicircular canal activation, increasing the FPE in supine conditioning and decreasing it in the prone position. This remarkable enhancement and reduction of the FPE suggests that asymmetric activation of the otolithic system induces an adaptive process similar to that occurring with asymmetric semicircular canal stimulation, enhancing the perception of fast rotation and reducing that of slow rotation after conditioning. The effect is reversed with respect to semicircular canal activation depending on how gravity modulates the otolithic receptors in prone and supine conditioning,

It is likely that the different effects between supine and prone conditioning depend on the activation polarity of the canals and otoliths. There is an increase in FPE in supine conditioning when rapid activation of the horizontal semicircular canal occurs in combination with rapid lateral (external) displacement of the otolithic mass of maculae on the same side. Conversely, there is a decrease in FPE in the prone position, where otolithic fast displacement is reversed with respect to canal activation (Figs. 2, 3). The enhancement of FPE in the supine condition is probably due to a further reduction of slow-motion perception induced by otolithic stimulation, while slow movement perception is increased in the prone position. We suggest that the sign of otolithic and canal stimulation is not congruous in the supine position since, in most natural horizontal rotation in combination with translation to one side, activation of the canal is combined with displacement of the otolithic mass on the same side towards the internal side. This incongruous activation may further reduce the sensitivity to slow movement perception. The reverse may occur in the prone position where the otolithic mass is displaced in the opposite direction.

Moreover, the different magnitudes of the additive and subtractive effects after supine and prone conditioning suggest that otolithic and canal signal interaction and adaptation do not add linearly, as previously shown for perceptual responses (Crane 2016; Crane and Demer 1997; Israel et al. 2005; Ivanenko et al. 1997; Soyka et al. 2015; MacNeilage et al. 2010).

Assessing this contribution to self-motion perception can be useful for examining otolithic function under normal and pathological conditions. In fact, reflex examination may be insufficient to explore the function of the otoliths since reflex and perception are differently centrally elaborated (Panichi et al, 2017a, b), especially when there is a full recovery of reflexes and subjects perceive instability and erroneous self-motion perception. It may be useful to evaluate the increase and decrease of the FPE after supine and prone conditioning to better highlight possible otolithic dysfunction, as, for example, in lithiasis of the semicircular canals (Faralli et al. 2011), in particular, when patient discomfort persists in the presence of normal reflex values.

In conclusion, the fairly simple test we propose can be very useful to reveal persistent vestibular dysfunction during examination in clinics. It can allow the perceptual otolithic contribution to be recognized, even with normal rotation. Four cycles of asymmetric rotation barely mimic real situations, but adaptation occurs even after the first cycle of oscillation as shown for the response to semicircular canals (Pettorossi et al, 2013). Therefore, the test of asymmetric rotation reveals a perceptual adaptive property in the function of otoliths that may operate normally when slow rotation follows fast rotation.
